# Sentinel node theory helps tracking of primary lesions of cancers of unknown primary

**DOI:** 10.1186/s12885-020-07042-6

**Published:** 2020-07-09

**Authors:** Yilin Shao, Xin Liu, Silong Hu, Yingjian Zhang, Wentao Li, Xiaoyan Zhou, Qifeng Wang, Yifeng Hou, Yong Chen, Yanli Wang, Yaohui Wang, Zhiguo Luo, Xichun Hu

**Affiliations:** 1 Department of Medical Oncology, Fudan University Shanghai Cancer Center, 270 Dong-an Rd, Shanghai, 200032 China; 2 Shanghai Medical College, Fudan University, Shanghai, 200032 China; 3 Department of Nuclear Medicine, Fudan University Shanghai Cancer Center, Shanghai, 200032 China; 4 Department of Pathology, Fudan University Shanghai Cancer Center, Shanghai, 200032 China

**Keywords:** Sentinel lymph node, Cancer of unknown primary, Diagnosis

## Abstract

**Background:**

Sentinel lymph node is the first stop of lymphatic spreading of cancer with known primary. The lymph node metastasis pattern of cancer of unknown primary (CUP) is unclear and has been presumed to follow the same pathway. To test this hypothesis, data of all 716 patients clinically diagnosed as CUP in our center were collected.

**Methods:**

Diagnoses of lymph node metastasis were established by ^18^F-FDG PET-CT and/or biopsy pathology. Three hundred and forty-seven cases meeting the criteria were divided into three groups: pathology-confirmed primary with invasive biopsy or surgery of the suspicious lesion (group A, *n* = 64), primary still unknown even with invasive biopsy or surgery of the suspicious lesion (group B, *n* = 204), and others with no suspicious lesion or lesions who had not been sampled due to medical or other reasons (group C, *n* = 79). We assessed the clinicopathological features between these groups, and the relationship between lymph node metastasis pattern and confirmed primary site.

**Results:**

In group A, the primary sites of 61 cases were compatible with sentinel node theory, resulting in a positive predictive value of 95%. No significant differences in age, sex, bone metastasis, or visceral metastasis observed between group A and group B, except that group A had a higher ratio of differentiated carcinoma (94% vs. 77%, *P* = 0.003).

**Conclusion:**

To our knowledge, this is the first evidence indicating that the majority of clinical CUP cases follow the sentinel node theory to spread in lymph nodes, which helps tracking the primary, especially for differentiated carcinoma.

## Background

Cancer of unknown primary (CUP) is characterized as a disease with early dissemination of metastases without a primary detected site after extensive laboratory and clinical investigations [1]. CUP accounts for 3–5% of all human cancers, reported to be the seventh to eighth most frequent malignant tumor, and the fourth most common cause of cancer death [2]. The diagnostic work-up of CUP mainly consists of two stages [3]: (1) general examination: thorough medical history and physical examination, basic blood and biochemistry analyses including serum tumor markers, computed tomography scans, and breast ultrasound or mammography (if needed); (2) special examination: breast magnetic resonance imaging, endoscopies, and additional diagnostic pathology including immunohistochemical staining (IHC) of pathology slides. However, there has been no international standard definition of CUP since diagnostic codes lack consensus [4]. In this study, clinical CUP was defined as histologically confirmed metastatic tumors with no identified primary site, or only a suspicious primary site identified following completion of the diagnostic work-up.

Clinical data about treatment of CUP are scarce and consensus guidelines have not been decided yet [5–8]. Recent study found that site-specific treatment based on microarray profiling did not improve 1-year survival compared with empirical chemotherapy [7], but this study has its own limitations such as the limited number of patients within the efficacy cohort [9]. Considering that site-specific treatment advancements have improved survival greatly during the past decade for CUP patients predicted to have common tumor types like breast cancer, much effort has been made to identify the primary site of CUP patients [9]. In the precision medicine era, new techniques such as microRNA assays, gene expression profiling, and DNA-methylation profiling were introduced for diagnosis of CUP [4], and may provide an opportunity to benefit from novel personalized therapies [10]. However, the accuracy of commercially available gene profiling assays for CUP is approximately 83–89% [4, 11], and these new techniques are rather expensive, unavailable in many districts, and require a sufficient amount of tumor sample. It also generates false-positive and false-negative data [11]. Even with all these methods, the ability to track the primary of CUP is still restricted.

It would be very helpful and convenient if we could determine the clinical clues that could guide the search of the primary sites of CUP patients. Our team has reported that oligo bone metastasis may suggest the primary site to be in the neighboring area in patients without visceral metastasis [12]. Lymphatic spreading is a relatively early process in distant metastasis of cancer and nodal metastasis of most primary-known solid tumors follows sentinel lymph node (SLN) theory. SLNs are the first stop of lymph nodes to receive drainage from the primary tumor. We know that lymphatic drainage from the primary tumor often travels to the SLNs first, and then sequentially to other regional lymph nodes. Gould et al. were the first to describe SLNs at the junction of the anterior and posterior facial vein in parotid cancer in 1960 [13]. Cabanas (1977) followed with a study in penile cancer, identifying SLNs at the junction of the femoral head and the ascending ramus of the pubis [14]. Morton and coworkers developed a technique for intraoperative mapping to selectively remove lymph nodes on the direct drainage pathway from primary melanomas, and SLNs were considered to be the first site of metastatic disease [15]. Then, they introduced blue dye-mapping of lymph nodes, which was a crucial point in the general acceptance of sentinel node biopsy [16]. Nowadays, sentinel node biopsy is routinely used for tumor staging and treatment especially in melanoma, breast cancer, and oral cancer [17].

In clinical practice, some CUP patients, such as with cervical lymph node metastasis of squamous-cell carcinoma and axillary lymph node metastasis of adenocarcinoma, are being treated as for head and neck cancer and breast cancer respectively, and have the favorable prognosis [1, 18]. However, there has been no solid evidence why we can do so. In this study, we hypothesize that the lymph node metastasis (LNM) pattern of CUP follows the same pathway as cancers with known primary sites to spread in the lymph nodes. To test this hypothesis, we analyzed the LNM pattern of patients with clinical CUP to determine if the SLN theory provides some clues in tracking the primary site of CUP. To our knowledge, there have been no other reports or data about this issue.

## Methods

### Study population

The inclusion criteria were as follows: (1) patients clinically diagnosed as CUP with histologically confirmed metastatic tumors whose primary site cannot be identified after standard pretreatment evaluation in Fudan University Shanghai Cancer Center between January 2006 and June 2018; (2) LNM diagnosed by one or both of the two means: lymph node biopsy with pathological results and ^18^F-FDG PET-CT scanning, which can assess both morphology and function of lymph nodes by measuring values of standard uptake values.

The exclusion criteria were as follows: (1) LNM with more than two directions of lymphatic drainage or with supraclavicular LNM only; (2) a history of previous malignancy except non-melanoma skin cancer or in situ carcinoma of the cervix.

We ruled out patients with supraclavicular LNM only because it was hard to figure out one most possible primary site as supraclavicular lymph nodes could be SLNs for a few types of cancer. We ruled out patients with more than two directions of lymphatic drainage also because it was hard to infer one most possible primary site of CUP in this instance.

### Diagnostic procedures

We collected the data of all 716 patients clinically diagnosed as CUP in our institute between January 2006 and June 2018. The diagnosis of LNM was separately made by two experienced doctors from the Department of Nuclear Medicine and/or Pathology. A third doctor was only invited when the two doctors were in disagreement. For lymph nodes with cancer involvement, our pathologists routinely selected some tissue-specific antibodies for immunostaining to determine the possible primary site. Afterwards, the attending physicians combined all the available information and decided to take sampling on which lesion. The “prime suspect” for primary site refers to the highly suspicious lesion in the images, upon which a definitive diagnosis of primary tumor cannot be done in the eyes of radiological experts.

A total of 347 cases meeting the criteria were included and then divided into three groups: primary pathologically confirmed by invasive biopsy or surgery of the suspicious lesion (group A, *n* = 64), primary still unknown even with invasive biopsy or surgery of the suspicious lesion (group B, *n* = 204), and others with no suspicious lesion or lesions and who had not undergone invasive biopsy or surgery (group C, *n* = 79). The evaluation was authorized by the Clinical Research Ethics Committee of Fudan University Shanghai Cancer Center, Fudan University. All of the participants in this evaluation were well informed about the details, and informed consent was acquired.

### Statistical analysis

In cases with a pathologically confirmed primary site (group A), we analyzed the relationship between the primary site and the distribution of LNM to determine whether they met the SLN theory.

The Chi-square test was performed to compare the differences in the basic clinicopathological characteristics among different groups of clinical CUP patients using SPSS statistical software (version 22.0). *P*-values less than 0.05 were considered statistically significant.

## Results

The clinicopathological features of 347 cases enrolled were analyzed. The median age of these patients was 55 years old, ranging from 24 to 88 years old. All patients had LNM and some had metastases to other sites such as bone (11%), liver (4%), lung (5%), brain (2%), soft tissue (3%), peritoneum (2%), and ovary (1%). The diagnosis of LNM was established by pathology only (25%), ^18^F-FDG PET-CT scanning only (6%) and both (69%). Overall, 75% of these patients underwent ^18^F-FDG PET-CT scanning.

**Fig. 1:**
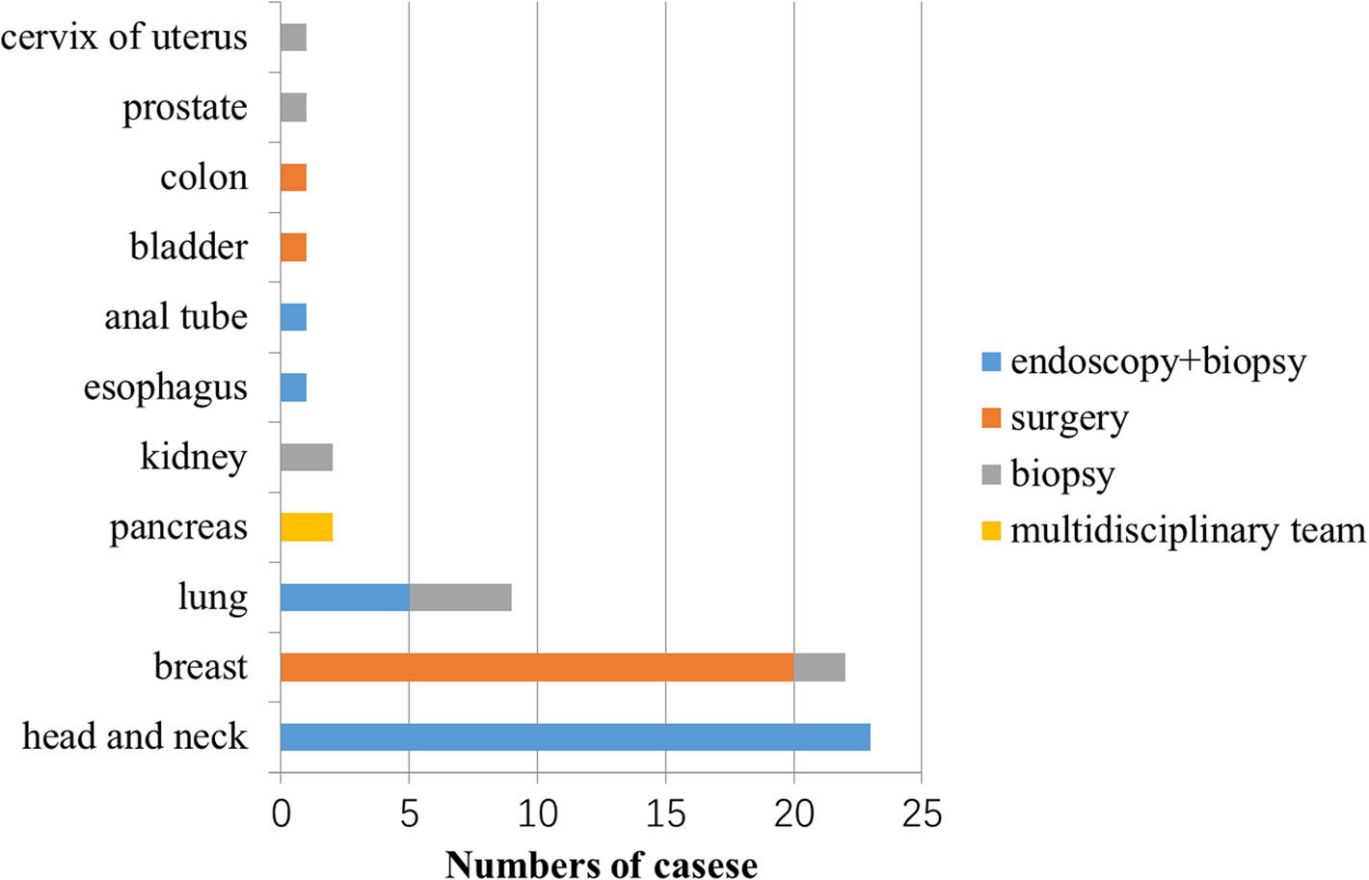
Methods used to establish a diagnosis of primary lesion

A total of 64 (18%, 64/347) patients had their primary tumor site identified (group A). The methods for identifying the primary sites are summarized in Fig. 1. Cases with suspicious head and neck cancer were all confirmed by endoscopy and biopsy (23/23 cases). Cases with suspicious breast lesions mostly underwent surgery and were confirmed by pathology (20/22 cases), and the other two cases underwent breast biopsy (2/22 cases). A total of 44% of cases with suspicious lung cancer were confirmed by lung aspiration biopsy (4/9 cases), while 56% of cases underwent bronchoscopy biopsy (5/9 cases). Two cases with suspicious pancreatic cancer were hard to biopsy, and the diagnosis of pancreatic cancer was made by the CMUP team and then confirmed by our pancreatic multidisciplinary team. Cases with suspicious renal cancer (2/2 cases), prostate cancer (1/1 case), and cervical cancer (1/1 case) were all confirmed by biopsy. Cases with suspicious lesions in the esophagus (1/1 case) and anal canal (1/1 case) were all confirmed by endoscopy and biopsy. Cases with suspicious lesions in the bladder (1/1 case) and colon (1/1 case) were all confirmed by surgery.

Among the 64 CUP patients in group A, we analyzed the relationship between LNM pattern and primary sites, as shown in Table 1. In 25 cases with only cervical LNM, the primary sites of 23 patients were located in the head and neck (92%), one in the cervical segment of the esophagus (also compatible with SLN theory) and one in the cervix (not compatible with SLN theory). In 22 cases with unilateral axillary lymph nodes with or without another one regional LNM, all patients turned out to be breast cancer. In 11 cases with lung hilar ± mediastinal lymph nodes with or without another one regional LNM, nine patients turned out to be lung cancer, one was kidney cancer, and one was prostate cancer (both not meeting the sentinel node theory). Two cases with retroperitoneal and celiac LNM both turned out to be pancreatic cancer. The primary site of one case with renal hilar and retroperitoneal LNM was the kidney, one case with peri-intestinal LNM was colon cancer, one case with peri-iliac vessels and inguinal LNM was from the anal canal, while one case with peri-iliac vessels, retroperitoneal and inguinal LNM was from the bladder. Overall, there were 61 cases compatible with SLN theory and three cases that were incompatible, leading to a positive predictive value of as high as 95% (61/64).

**Table 1:** Lymph node metastasis pattern of cancer of unknown primary and compatibility with sentinel node theory(*N* = 64)

Lymph node distribution	Primary tumor site	N	Compatibility
Cervical	Head and neck	23	
	Nasopharynx	5	Yes
	Oropharynx	5	Yes
	Parotid	4	Yes
	Sinus piriformis	3	Yes
	Root of tongue	3	Yes
	submandibular	2	Yes
	gland	1	Yes
	larynx	1	Yes
	Esophagus (cervical segment)	1	Yes
Cervical+supraclavicular	Cervical cancer of the uterus	1	No
Axillary	Breast	19	Yes
Axillary+supraclavicular	Breast	1	Yes
Axillary+internal mammary	Breast	1	Yes
Axillary+deep surface of pectoralis minor muscle	Breast	1	Yes
Lung hilar±mediastinal	Lung	4	Yes
Lung hilar±mediastinal +supraclavicular	Lung	2	Yes
Lung hilar±mediastinal+cervical	Lung/Prostate	2/1	Yes/No
Lung hilar±mediastinal+inguinal	Lung	1	Yes
Mediastinal+supraclavicular+lateral thoracic	Kidney	1	No
Retroperitoneal+celiac	Pancreas	2	Yes
Renal hilar+retroperitoneal	Kidney	1	Yes
Peri-intestinal	Colon	1	Yes
Peri-iliac vessels+inguinal	Anal canal	1	Yes
Peri-iliac vessels+inguinal +retroperitoneal	Bladder	1	Yes

**Fig. 2:**
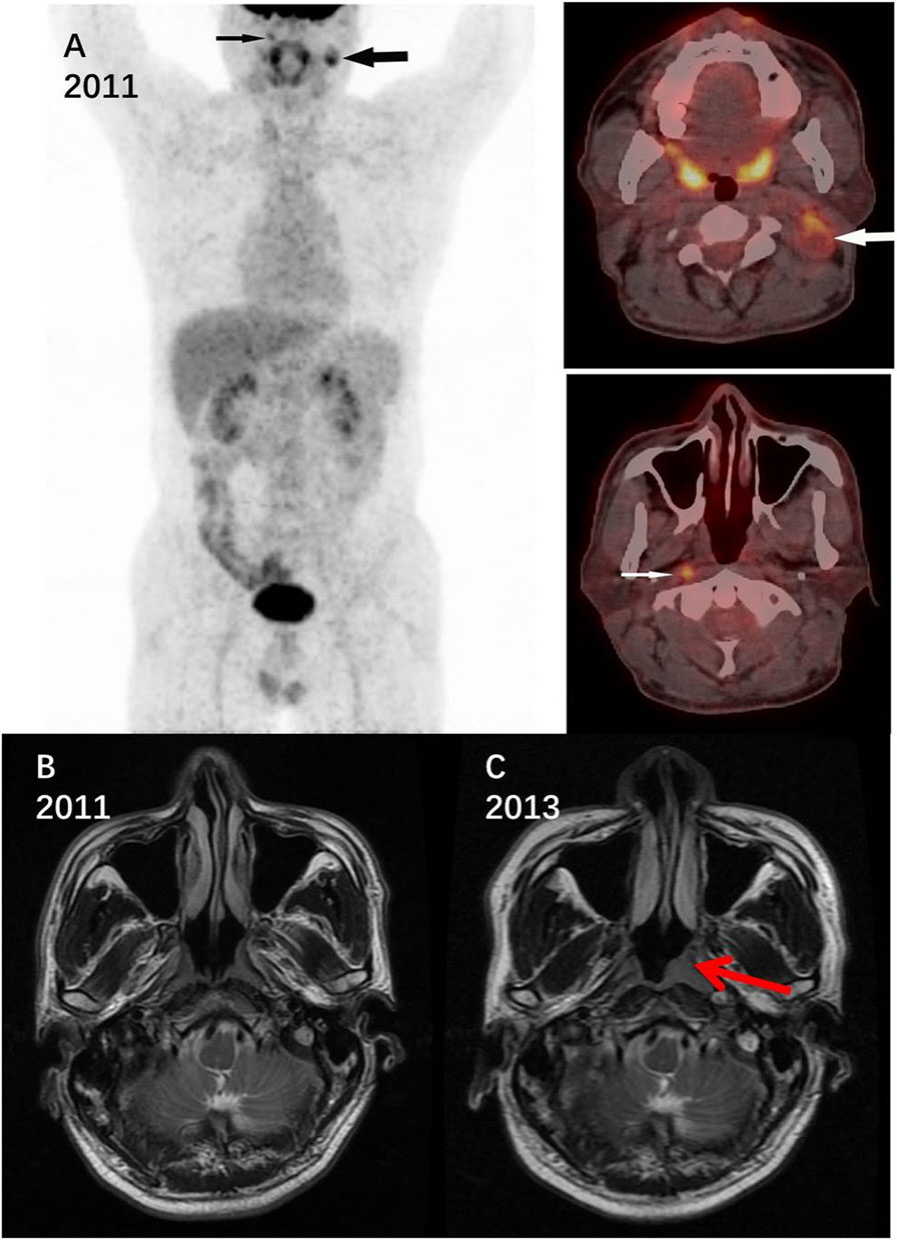
Images of a clinical CUP patient with cervical lymph node metastases. Nasopharynx endoscopy was negative in 2011, with the primary lesion emergent in 2013. The primary cancer was finally pathologically confirmed to be nasopharyngeal cancer, demonstrating the value of SLN theory in tracking the primary. A: Image of 18F-FDG PET-CT scan in 2011 showing only cervical lymph node metastases (black and white arrow) and was otherwise normal. B: Magnetic resonance imaging of nasopharynx in 2011 with no suspicious lesion. C: Magnetic resonance imaging of nasopharynx in 2013 with a suspicious lesion (red arrow), which was later confirmed by biopsy and pathological data

**Fig. 3:**
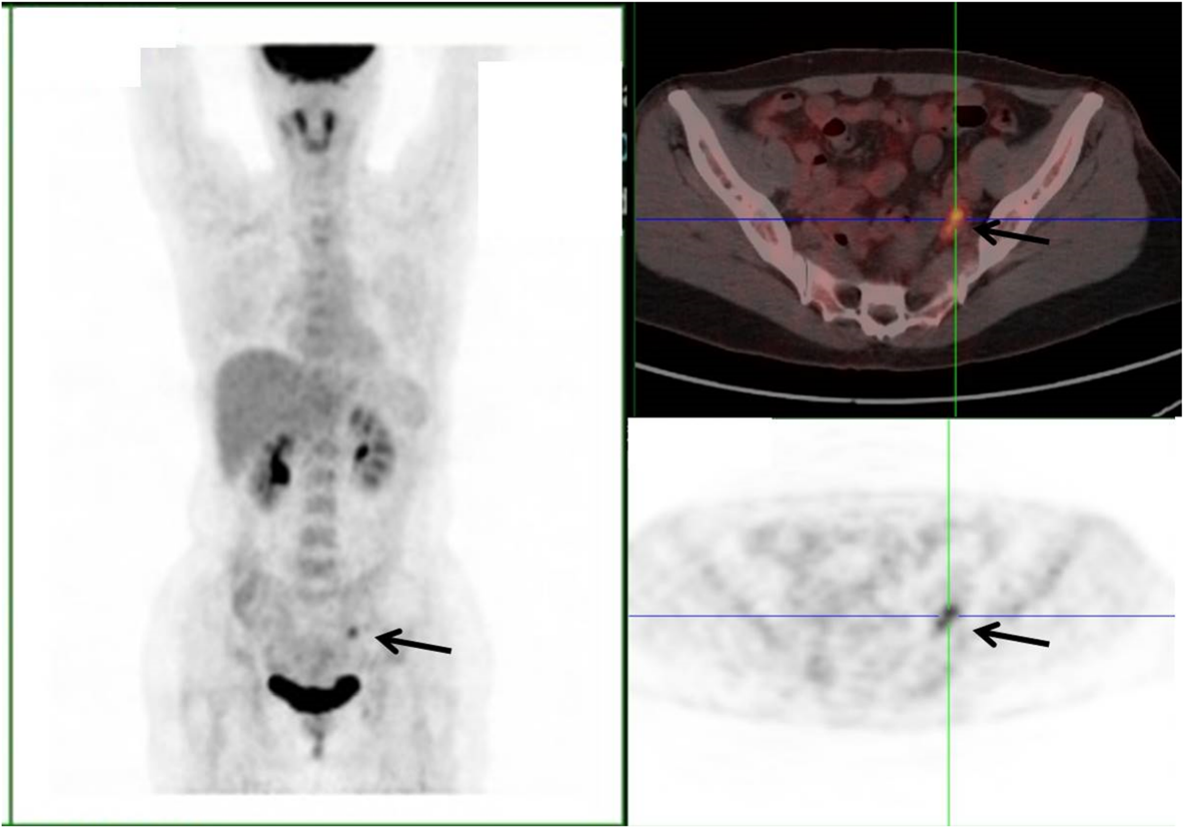
Images of a CUP patient with left iliac vessels and left inguinal lymph node metastasis. The patient had no symptoms such as diarrhea, constipation, or hematochezia. 18F-FDG PET-CT images after left inguinal lymph node surgery showed metastasis in a lymph node lining the iliac vessels identified (black arrow), and he was otherwise normal. Repeat anorectal examination showed a suspicious lesion in the anal canal, which was finally pathologically confirmed by enteroscopy, although the prior enteroscopy showed no abnormal findings

Figure 2 shows images of a representative CUP patient with no suspicious primary lesion and only cervical LNM in 2011. Endoscopy of the nasopharynx was negative. Two years later, the primary site was found at the nasopharynx, and was pathologically confirmed to be nasopharyngeal cancer. Figure 3 shows images of ^18^F-FDG PET-CT of a patient with left peri-iliac vessels and left inguinal LNM who was diagnosed as clinical CUP. Based on the SLN theory, the CMUP team considered the prime suspect to be in ipsilateral extremity or pelvic region. Anorectal examination was done again and found a suspicious lesion in the anal canal. Further enteroscopy did a biopsy of the lesion, giving a definitive diagnosis of anal cancer, although the prior enteroscopy showed no abnormal finding.

Overall, there were 64 patients in group A, 204 patients in group B, and 79 patients in group C. Patients in group C did not undergo invasive biopsy or surgery, so they may have had an undetected potential primary site. Therefore, we only analyzed clinicopathological features between patients in group A and group B. As shown in Table 2, patients in group A and group B had no significant difference in age (*P* = 0.679), sex (*P* = 0.956), bone metastasis (*P* = 0.330), and visceral metastasis (*P* = 0.073), suggesting that CUP had similar clinical features between cases with confirmed and unconfirmed primary sites. However, cases in group A had a significantly higher percentage of differentiated carcinoma than group B (94% vs. 77%, *P* = 0.003).

**Table 2:** Clinical and pathological features of clinical CUP patients who had undergone histopathological examinations of suspicious primary lesions

	Group A Primary site confirmed (*N* = 64)	Group B Primary site not confirmed (*N* = 204)	*P* value
Age, years (x ± s)	54.47 ± 9.73	55.10 ± 10.98	0.679
Sex			0.956
Male	31(48%)	98(48%)	
Female	33(52%)	106(52%)	
Bone metastasis	6(9%)	12(6%)	0.330
Visceral metastasis	9(14%)	14(7%)	0.073
SCC	26(41%)	66(32%)	0.224
Non SCC	38(59%)	138(68%)	
Adenocarcinoma	34(53%)	91(45%)	0.233
Non adenocarcinoma	30(47%)	113(55%)	
Poor- or un-differentiated	4(6%)	47(23%)	0.003*
Differentiated	60(94%)	157(77%)	

*SCC* Squamous cell carcinoma. *P values less than 0.05 were considered statistically significant

## Discussion

For majority of cancers, primary lesions are usually identified first, followed by LNM or distant metastases. In patients with CUP, the metastatic lesions are identified first, and then attempts are made to track the primary lesion. In our study, as high as 95% of the pathology-confirmed primary sites in patients with clinical CUP were located at the drainage area of LNM, which first demonstrated that SLN theory is helpful in tracking the primary site of CUP. Based on this, a potential candidate primary lesion that could metastasize to the lymph nodes should be extensively investigated by thorough physical or radiological examination, or even biopsy. To our knowledge, there has been no previous report or published data focusing on this issue.

CUP was once viewed almost as a unique type of cancer. Now it is believed that most CUPs have primary sites and probably retain the gene signature of the putative primary origin [5]. Determining the primary site or the tissue-of-origin may have a substantial effect on therapeutic approaches as well as patient survival. However, most prior efforts have focused on IHC staining or gene expression profiling to determine the possible tissue of origin, and these approaches have problems such as high cost, the requirement for sufficient tumor tissue, and patients no longer being treatment candidates by the time results become available [19]. Moreover, they are unavailable in many districts, and can produce false-positive and false-negative data.

^18^F-FDG PET-CT is also a valuable diagnostic tool for patients with CUP. One meta-analysis showed that the overall primary tumor detection rate, pooled sensitivity, and specificity of ^18^F-FDG PET-CT were 37, 84, and 84% respectively [20]. Several studies found that ^18^F-FDG PET-CT could detect the occult primary tumor in as high as 49–57% of CUP cases [21–23]. The factors limiting the use of ^18^F-FDG PET-CT include its high cost and its limited value in small size tumor and tumors exhibiting a low FDG uptake [24]. One recent study by Cengiz et al. showed that ^18^F-FDG PET-CT does not represent a clear diagnostic advantage over conventional imaging methods regarding the ability to detect the primary tumor site [25, 26].

Therefore, radiological examination, pathological features, and molecular profiling are still not adequate for tracking of the primary site of CUP. Our study shows that as many as 95% of the confirmed primary sites in CUP cases are consistent with SLN theory. Clinical use of SLN theory includes cooperation between clinical oncologists and diagnostic experts to systemically review all available clinical information, to identify clues to indicate potential primary lesions, and then to undertake biopsy or operation on the prime suspect for pathological diagnosis. With this multidisciplinary approach, the tracking of primary lesions of CUP could be much improved.

Numerous studies have confirmed that SLNs are the first stop reached by metastatic cancer cells as they enter the regional lymphatic basin in the vast majority of cancer patients [16]. SLN theory has been widely used in surgery as SLN biopsy, resulting in fewer axillary lymph node dissections and fewer lymphedema cases, and thus improved quality of life [27–30]. Therefore, SLN theory has been of vast assistance in nodal staging and treatment options. However, to our knowledge, there has been no study to assess the role of SLNs in tracking the primary of CUP. In this study, we determined the compatibility of SLN theory according to previous studies. For example, previous studies showed that the sentinel lymph nodes of head and neck cancer were mostly cervical lymph nodes [31]. Therefore, when we found a CUP patient with cervical lymph node metastasis with or without supraclavicular lymph node metastasis, as they were in the same direction of lymphatic drainage, we assumed the primary site of this patients lying in the head and neck according to SLN theory. Besides, axillary nodes were often thought to be SLNs of breast cancer [27, 32], SLNs of lung cancer were often believed to be hilar or mediastinal nodes [33], and inguinal lymph nodes were believed to be SLNs of cancer in pelvic cavity like urologic cancer or anal canal cancer [34, 35]. We assumed the potential primary site in the same way according to SLN theory and compare it with pathologic results.

Early dissemination, aggressiveness, and unpredictable metastatic patterns are characteristic of CUP and it has been recommended that physicians should not rely on patterns of metastases to determine the primary site. However, a study by Hemminki et al. suggested that location of metastasis may predict site-specific cancer deaths and provide insights into the location of primary tumors [36]. SLN theory has also been used in some cases of CUP. For example, adenocarcinoma in axillary lymph nodes in women are often presumed to originate from breast cancer [37], consistent with our findings.

To our knowledge, this is the largest sample size study showing that in clinical CUP patients, tumor grading affects the final identification of primary lesions, with a statistically significant difference of 94% vs. 77% for differentiated vs. un- or poorly-differentiated tumors, respectively. Previously, Peter et al. found that sinonasal undifferentiated carcinoma had higher rates of nodal involvement than sinonasal small-cell carcinoma [38]. Another study showed that patients with undifferentiated carcinoma had higher rates of advanced stage disease than patients with endometrioid adenocarcinoma [39]. These findings suggested that undifferentiated carcinoma was more likely to undergo early metastasis, making it harder to identify the primary site in CUP.

Besides the largest sample size, another strength is that 75% patients had ^18^F-FDG PET-CT scanning, providing a systemic review of cancer status both in and outside the lymph nodes for each patient. However, our study also has its own limitations. First, most cases did not receive second opinions from the CMUP multidisciplinary team. However, all our patients were diagnosed and treated following our institute’s guidelines or rules. Second, the diagnosis of LNM in 6% of patients was established by ^18^F-FDG PET-CT only. However, the accuracy of ^18^F-FDG PET-CT in predicting axillary LNM was as high as 78–95% [40], and it detected abnormal lymph nodes more often than CT [41]. Therefore, ^18^F-FDG PET-CT is the best available method integrating the structure and function of lymph nodes. Third, the number of identified primary site of CUP cases with abdominal or pelvic LNM were fewer than those with cervical or axillary LNM. The possible reasons are: (1) LNM in abdominal or pelvic cavity are deep and hard to find, so there tend to be more than two directions of lymph drainage, making it less likely that potential primary lesions can be located. (2) CUP patients with cervical lymph node or axillary LNM are favorable types and have better prognosis, allowing more time for the primary lesion to be detected [1].

In this study, we divided CUP patients into three groups. Group A included patients of pathology-confirmed primary with invasive biopsy or surgery of the suspicious lesion, and in this group, we were able to verify SLN theory in majority of patients (95%). Group B included patients of primary still unknown even with invasive biopsy or surgery of the suspicious lesion. As there was no statistically significant difference between group A and group B in terms of clinical and pathological features, except that cases in group A had a significantly higher percentage of differentiated carcinoma than group B, we thought it is reasonable to extrapolate the findings of group A to group B, or at least for differentiated CUP. Group C was a more complex group including patients with no suspicious lesion or lesions had not been sampled due to medical or other reasons. This means group C may also include potential patients in group A or B if they got sampled. Therefore, patients in group C need more aggressive examination for further study, and this is also one of the limitations in our study. In conclusion, it is reasonable to extrapolate the findings of group A to group B, and possibly to group C, or at least for differentiated CUP. Therefore, our investigation showed that the majority of CUP cases followed the SLN theory.

## Conclusions

In conclusion, our study first shows that the majority of CUP cases follow SLN theory which comes from cancer of known primary to spread in lymph nodes, indicating its value in the tracking of primary lesions of CUP. Furthermore, primary sites are more likely to be identified in CUP patients with differentiated carcinoma.

## Data Availability

The datasets used and/or analysed during the current study are available from the corresponding author on reasonable request.

## Abbreviations

CUP: Cancer of unknown primary
IHC: Immunohistochemical staining
SLN: Sentinel lymph node
LNM: Lymph node metastasis
